# Prediagnostic serum iodine and selenium in relation to breast cancer survival

**DOI:** 10.1093/bjsopen/zrag047

**Published:** 2026-06-16

**Authors:** Magdalena Szramka, Malte Sandsveden, Kamil Demircan, Signe Borgquist, Ann Rosendahl, Jonas Manjer

**Affiliations:** Department of Clinical Sciences Malmö, Lund University, Malmö, Sweden; Department of Surgery, Skåne University Hospital, Malmö, Sweden; Department of Clinical Sciences Malmö, Lund University, Malmö, Sweden; Department of Surgery, Vrinnevisjukhuset, Norrköping, Sweden; Precision Healthcare University Research Institute, Queen Mary University of London, London, UK; Computational Medicine, Berlin Institute of Health at Charité—Universitätsmedizin Berlin, Berlin, Germany; Department of Oncology, Aarhus University Hospital, Aarhus University, Aarhus, Denmark; Department of Clinical Sciences Lund, Oncology, Lund University, Skåne University Hospital, Lund, Sweden; Department of Clinical Sciences Malmö, Lund University, Malmö, Sweden; Department of Surgery, Skåne University Hospital, Malmö, Sweden

**Keywords:** epidemiology, mortality, elements

## Abstract

**Background:**

Trace elements like iodine have been suggested to be associated with breast cancer due to their antioxidant properties and ecological differences in incidence and mortality. Yet, there are no epidemiological studies on breast cancer prognosis in relation to iodine levels. The primary aim of this study was to investigate the association between prediagnostic serum iodine levels and breast cancer survival. A secondary aim was to examine if the potential association was modified by serum selenium levels.

**Methods:**

This study included women diagnosed with primary invasive breast cancer (1991–2013) from the Malmö Diet and Cancer Study (1991–1996). The patients were divided into quartiles depending on their serum iodine levels at baseline. Crude and multivariable-adjusted Cox regression analyses were used to obtain hazard ratios with 95% confidence intervals, for breast cancer-specific and overall mortality, with follow up until 31 December 2018. Analyses were additionally stratified by selenium levels (below *versus* above the median).

**Results:**

Out of 17 035 female participants of the Malmö Diet and Cancer Study, 918 patients with breast cancer were included in this study. The mean(standard deviation) follow-up time was 11.4(6.0) years. The adjusted hazard ratios for breast cancer-specific and overall mortality for the highest iodine quartile compared with the lowest were 1.26 (95% confidence interval 0.79 to 1.99) and 1.27 (0.92 to 1.75). Among women with high selenium, high iodine levels were associated with a high breast cancer-specific and overall mortality; hazard ratios of 1.90 (1.09 to 3.32) and 1.81 (1.26 to 2.61), respectively. High selenium levels were associated with a lower breast cancer-specific and overall mortality; the adjusted HRs were 0.69 (0.50 to 0.95) and 0.73 (0.58 to 0.91).

**Conclusion:**

High serum iodine was weakly associated with mortality overall and significantly associated among women with high selenium, whereas serum selenium alone was inversely associated with mortality.

## Teaser

Iodine and selenium have been suggested to be associated with breast cancer due to their antioxidant properties and geographical differences in incidence and mortality. This study investigated prediagnostic serum iodine and selenium levels in relation to breast cancer survival and found that iodine had a weak association with mortality, selenium alone had an inverse association, but high iodine with high selenium was a significant risk factor for a worse prognosis.

## Introduction

Iodine has been suggested to be protective against breast cancer development and progression in experimental studies^1,2^. A recent study^3^ has shown that high prediagnostic iodine levels are associated with a lower breast cancer risk, but there are no studies on the association between iodine and prognosis.

Iodine is an essential trace element abundant in fish, seaweed, dairy products, and iodinated salt. Japan and Korea are known to have low breast cancer incidence and mortality rates and it has been suggested that this is related to a high seaweed intake^4^.

In clinical studies, supplementation with iodine compositions has demonstrated a reduction in mastalgia and fibrocystic breast disorders, suggesting its potential role in breast physiology^5^. Furthermore, radioiodine uptake has been demonstrated in the alveoli and ductules of non-lactating mammary glands^6,7^. Animal studies have previously shown that a state of iodine deficiency induces morphological changes resembling atypia in the human breast.

A study^3^ from Sweden recently demonstrated a 25% lower risk of breast cancer in women with high serum iodine compared with low levels, whilst simultaneously having high selenium levels. It has been proposed that the two elements interact in a synergistic manner through selenoenzymes and that they could have a local effect in breast cancer development and/or progression^4^.

In terms of breast cancer prognosis, there are no studies on serum iodine concentrations and breast cancer recurrence and mortality. However, preclinical studies have shown an inhibitory effect on cancer cell proliferation by the administration of iodine containing compounds in both *in vitro* and *in vivo* studies^8–10^. A recent randomized pilot study^11^ among chemotherapy-treated patients with breast cancer demonstrated that oral supplementation of iodine had an inhibitory effect on tumour progression, a reduction in side effects of chemotherapy, and an increased survival time following treatment.

The aim of this prospective study, as the first of its kind, was to investigate the association between prediagnostic levels of serum iodine and breast cancer survival, as measured by breast cancer specific and overall mortality. Additionally, a potential interaction between iodine and selenium in relation to mortality was investigated.

## Methods

### The Malmö Diet and Cancer Study (MDCS) and study population

The MDCS is a prospective population-based cohort, a part of the European Prospective Investigation into Cancer and Nutrition Cohort^12^. Data were collected between the years 1991 and 1996 through a questionnaire regarding lifestyle factors, reproductive health, and medication. Basic measurements of weight and height were documented. Blood samples were drawn and stored at −80°C until the analyses. Women who had any of the following were excluded from the study population: breast cancer at baseline, inadequate amount of blood sample, extremely high iodine values described previously by Manjer 2020^3^, carcinoma i*n situ*, and bilateral breast cancer. The statistical software SPSS v25 (IBM® SPSS® Statistics for Windows, v25 (IBM Corp., Armonk, NY, USA)) was used for the analyses.

The present study was approved by the ethics committee in Lund (Dnr 2015/283). Written informed consent was obtained from all study participants at baseline to enable the collection of samples and information; this includes future follow-up (original ethical approval: LU 51–90). Follow-up studies have been advertised in local media, and information has been given about the option to withdraw.

### Laboratory analyses

Serum iodine and selenium concentrations were analysed from the samples by ALS Scandinavia AB (Luleå, Sweden) using single-element standard solutions, traceable to the National Institute of Standards and Technology, on an Inductively Coupled Plasma Sector Field Mass Spectrometry instrument (ICP-SFMS, Thermo Element 2). Samples were prepared by diluting 0,15 ml serum in an alkaline solution containing 0.1% NH3 and 0.005% EDTA/Triton-X to obtain a final 10 ml solution. Seronorm, Trace Elements Serum (Lot 0608414, SERO AS, Norway) was used as reference material for accuracy control and analysed within each assay batch.

### Tumour characteristics

Data on tumour characteristics were collected by different methods during three different time periods. Information on tumour size, lymph node involvement, and distant metastasis at diagnosis was obtained from medical records for all periods. Histological grade was determined by re-examinations of the tumour material from patients diagnosed between 1991 and 2004 and collected from medical records for the tumours diagnosed from 2005 and onward.

Oestrogen receptor (ER) and progesterone receptor (PgR) status was evaluated by immunohistochemical assessment (IHC) on tissue microarrays (TMAs) on the tumours diagnosed between 1991 and 2004 and were considered positive when > 10% of the nuclei were stained. From 2005 and onward, medical records were used to collect data on hormone receptor status.

Human epidermal growth factor receptor (HER2) status was obtained primarily from National Registries data. When the data were not available, clinical records were used. If the information was missing, TMAs were assessed for HER2-expression for the completion of the data, but only up until 2004. Thereafter, clinical records were used. HER2 was considered positive if *in situ* hybridization (ISH) was amplified or, where data on ISH were not available, immunohistochemistry was graded as 3 +. Where no ISH was available and IHC was graded 2 +, HER2 was classified as missing. All other results were classified as negative for HER2.

Ki67, a nuclear marker of proliferation, was evaluated by TMA from 1991 to 2007 and divided according to internal rank into three categories as low, intermediate, and high^13^. Thenceforth, clinical records were used to obtain the data, as for the other tumour characteristics.

Surrogate intrinsic types used in the descriptive analysis conform to local standards proposed by the South Swedish Breast Cancer Group and were created for a previous study^13,14^. The intrinsic groups are classified accordingly: Luminal A-like; Luminal B-like, HER2 overexpressing, and triple negative breast cancers, and have been described in more detail elsewhere^13^.

### Follow-up

The Swedish Cause of Death Registry was used to collect data on mortality by record linkage using the Swedish Personal Identity number, a unique number for each resident of Sweden. Endpoint data were collected up until the end of follow-up on 31 December 2018. Breast cancer-related death was defined as breast cancer being the underlying or contributing cause of death. All patients who were not censored due to death or emigration were followed until the endpoint.

The patients who emigrated during the study were censored at the date of emigration as given by the Swedish Tax Agency. All registries mentioned can be cross-linked through the unique Swedish citizen personal number.

### Statistical methods

The cohort was divided into quartiles (Q) according to serum iodine and selenium concentrations. The quartiles were cross tabulated with tumour characteristics and factors known to affect prognosis such as ER status, PgR status, HER2 status, Ki67, tumour size, lymph node status, distant metastases, histological grade, and age at diagnosis^15,16^.

Breast cancer-specific mortality and overall mortality in different quartiles were compared using Kaplan–Meier plots. Corresponding mortality rates were calculated per 100 000 person years. Iodine levels and their association with mortality were investigated using a Cox proportional hazards model yielding hazard ratios (HRs) with 95% confidence intervals (c.i.). The analysis was subsequently adjusted for established tumour characteristics known to affect prognosis, as specified above. A subsequent analysis was conducted examining iodine and mortality in different strata of serum selenium levels. Selenium levels were dichotomized into categories of high and low using the median. Another analysis was performed comparing breast cancer mortality between women with high and low selenium concentrations, and these analyses were stratified for iodine. The analyses were adjusted for the same prognostic factors as previously mentioned. Interaction analyses were performed for the dichotomized selenium and iodine levels by inserting a multiplicative term in the Cox model, which is presented if *P* for interaction < 0.05. Missing information in covariables was handled as a separate category in the multivariable analyses. The information on stage, that is, tumour size, lymph node status, and distant metastasis, was complete to more than 90% for each of these variables. A sensitivity analysis was included where patients with missing information on tumour size were excluded.

Apart from analysing iodine as a categorical variable, it was also analysed in the main analysis as a continuous variable. Iodine values were standardized into z-scores, thus yielding HRs per standard deviations (s.d.). To investigate potential non-linear associations, Cox proportional hazards models with iodine as a continuous variable were augmented with restricted cubic splines (three knots at 10th, 50th, and 90th centiles) to allow for non-linearity. Non-linear analyses were conducted for both all-cause and breast cancer-specific mortality in the whole cohort. To assess non-linearity formally, restricted cubic splines models were compared with nested linear models using the likelihood ratio test. *P* < 0.05 was considered to depict associations significantly deviating from linearity. Restricted cubic splines models were visualized. R (version 4.3.1) with Rstudio was used to conduct the analyses. The packages rms (version 6.7.1) was utilized to compute restricted cubic splines and ggplot2 (version 3.5.2) was used to visualize the models.

There is a potential degrading of samples during storage, and a sensitivity analysis was performed for the main analyses using quartiles, adjusting for the period between when the blood samples were drawn and the time of the analysis for the present study.

## Results

In all, 28 098 participants between the ages of 44 and 74 were recruited for the MDCS, resulting in a participation rate of 40.8%. There were 17 035 females, of whom 576 had a breast cancer diagnosis at base line. When further exclusion criteria were applied (blood sample issues (130), extremely high iodine values (27), carcinoma i*n situ* (100), and bilateral breast cancer (20)), 918 cases of invasive breast cancer remained for the analysis of this study. The total number of incident breast cancer up until 31 December 2013 was 1762.

The inter-batch coefficient of variation was 4% for iodine and 3% for selenium and the detection limit was 2 µg/l and 4 µg/l, respectively.

Tumour characteristics in relation to iodine quartiles are presented in *Table 1*. Patients with high iodine levels were slightly older at baseline compared with patients with low levels (Q4 *versus* Q1), as well as more frequently diagnosed with smaller tumours and a histological grade I–II, but more often had lymph node metastases. Overall differences between iodine quartiles regarding established prognostic factors were small. There was no suggestion of relatively more missing information in any quartile.

**Table 1:** Prognostic factors and age distribution in relation to iodine quartiles

	Q1 *n* = 231	Q2 *n* = 227	Q3 *n* = 231	Q4 *n* = 229	Total *n* = 918
Iodine levels (μg/l), mean	< 60	≥ 60–< 68	≥ 68–< 78	≥ 78	73.45
Age at baseline (years), mean	54.9	55.7	56.8	57.1	56.1
Age at diagnosis (years), mean	66.1	66.1	66.5	66.6	66.3
**Tumour size (mm)**					
≤ 10	21.2%	21.1%	24.7%	24.9%	23.0%
>10−≤ 20	43.7%	44.9%	42.4%	45.9%	44.2%
>20−≤ 50	12.1%	6.2%	10.4%	6.6%	8.8%
> 50	13.9%	19.4%	13.9%	17.0%	16.0%
Missing	9.1%	8.4%	8.7%	5.7%	8.0%
**Histological grade**					
I	23.8%	25.1%	22.5%	24.9%	24.1%
II	44.6%	43.6%	41.1%	45.4%	43.7%
III	23.4%	23.8%	28.1%	20.5%	24.0%
Missing	8.2%	7.5%	8.2%	9.2%	8.3%
**KI67**					
Low	30.3%	27.8%	28.6%	27.5%	28.5%
Intermediate	20.3%	20.7%	22.9%	25.3%	22.3%
High	19.0%	24.7%	20.8%	20.5%	21.2%
Missing	30.3%	26.9%	27.7%	26.6%	27.9%
**Lymph node metastasis**					
0	64.9%	64.3%	62.8%	55.9%	62.0%
1–3	19.0%	14.5%	19.5%	21.4%	18.6%
4–9	3.9%	6.2%	5.6%	9.6%	6.3%
≥ 10	3.0%	5.7%	4.3%	1.3%	3.6%
Missing	9.1%	9.3%	7.8%	11.8%	8.4%
**Distant metastasis at diagnosis**					
No	91.3%	93.0%	91.3%	93.0%	92.2%
Yes	1.3%	0.9%	1.7%	1.7%	1.4%
Missing	7.3%	6.1%	6.9%	5.2%	6.5%
**Oestrogen receptor**					
Negative	7.4%	10.1%	10.8%	7.9%	9.0%
Positive	81.4%	77.5%	74.5%	77.7%	77.8%
Missing	11.3%	12.3%	14.7%	14.4%	13.2%
**Progesterone receptor**					
Negative	31.2%	34.4%	32.0%	29.3%	31.7%
Positive	55.0%	49.8%	50.2%	51.5%	51.6%
Missing	13.9%	15.9%	17.7%	19.2%	16.7%
**HER2**					
Negative	74.5%	74.4%	73.6%	71.6%	73.5%
Positive	7.8%	9.3%	7.4%	5.7%	7.5%
Missing	17.7%	16.3%	19.0%	22.7%	19.0%
**Intrinsic subtype**					
Luminal A-like	39.4%	40.1%	38.1%	41.5%	39.8%
Luminal B-like	19.9%	18.5%	18.2%	16.6%	18.3%
HER2 positive	7.8%	9.3%	7.4%	5.7%	7.5%
Triple negative	6.1%	7.0%	7.8%	5.2%	6.5%
Missing	26.8%	25.1%	28.6%	31.0%	27.9%

Values are % unless otherwise stated. HER2, human epidermal growth factor receptor.

In this cohort, prognostic factors such as histological grade, hormone receptor status, tumour size, and metastatic spread had the expected distribution in relation to the studied endpoints, that is, breast cancer death and overall mortality (*Table 2*). Mean(s.d.) time from date of diagnosis until endpoint was 11.4(6.0) years. Four cases had emigrated, and one case was diagnosed at the time of death, thus contributing with survival time accordingly.

**Table 2:** Prognostic factors and age distribution in relation to vital status

	Alive	Breast cancer death	Other cause of death	Total
	*n* = 578	*n* = 166	*n* = 174	*n* = 918
Iodine levels (µg/l), mean	72.92	75.04	73.72	73.45
Age at baseline (years), mean	53.9	58.3	61.2	56.1
Age at diagnosis (years), mean	65.0	66.9	70.2	66.3
**Tumour size (mm)**				
≤ 10	28.4%	5.4%	21.8%	23.0%
> 10−≤ 20	48.1%	31.9%	43.1%	44.2%
> 20−≤ 50	8.3%	10.8%	8.6%	8.8%
> 50	11.1%	31.3%	17.8%	16.0%
Missing	4.2%	20.5%	8.6%	8.0%
**Histological grade**				
II	45.8%	31.9%	47.7%	43.7%
III	18.5%	43.4%	23.6%	24.0%
Missing	6.6%	15.7%	6.9%	8.3%
**KI67**				
Low	33.0%	17.5%	24.1%	28.5%
Intermediate	20.8%	20.5%	29.3%	22.3%
High	17.8%	30.7%	23.6%	21.2%
Missing	28.4%	31.3%	23.0%	27.9%
**Lymph node metastasis**				
0	69.7%	34.3%	62.6%	62.0%
1–3	17.5%	24.1%	17.2%	18.6%
4–9	3.6%	16.9%	5.2%	6.3%
≥ 10	1.4%	12.0%	2.9%	3.6%
Missing	0.9%	4.2%	1.7%	1.6%
**Distant metastasis at diagnosis**				
No	95.3%	83.1%	90.2%	92.2%
Yes	0%	7.8%	0%	1.4%
Missing	4.7%	9.0%	9.8%	6.5%
**Oestrogen receptor**				
Negative	8.3%	12.7%	8.0%	9.0%
Positive	81.5%	60.8%	81.6%	77.8%
Missing	10.2%	26.5%	10.3%	13.2%
**Progesterone receptor**				
Negative	29.4%	38.6%	32.8%	31.7%
Positive	57.3%	31.9%	51.7%	51.6%
Missing	13.3%	29.5%	15.5%	16.7%
**HER2**				
Negative	78.0%	61.4%	70.1%	73.5%
Positive	6.1%	10.2%	9.8%	7.5%
Missing	15.9%	28.3%	20.1%	19.0%
**Intrinsic subtype**				
Luminal A-like	46.5%	18.1%	37.9%	39.8%
Luminal B-like	16.8%	24.1%	17.8%	18.3%
HER2 positive	6.1%	10.2%	9.8%	7.5%
Triple negative	5.7%	10.2%	5.7%	6.5%
Missing	24.9%	37.3%	28.7%	27.9%

Values are % unless otherwise stated. HER2, human epidermal growth factor receptor.

**Fig. 1:**
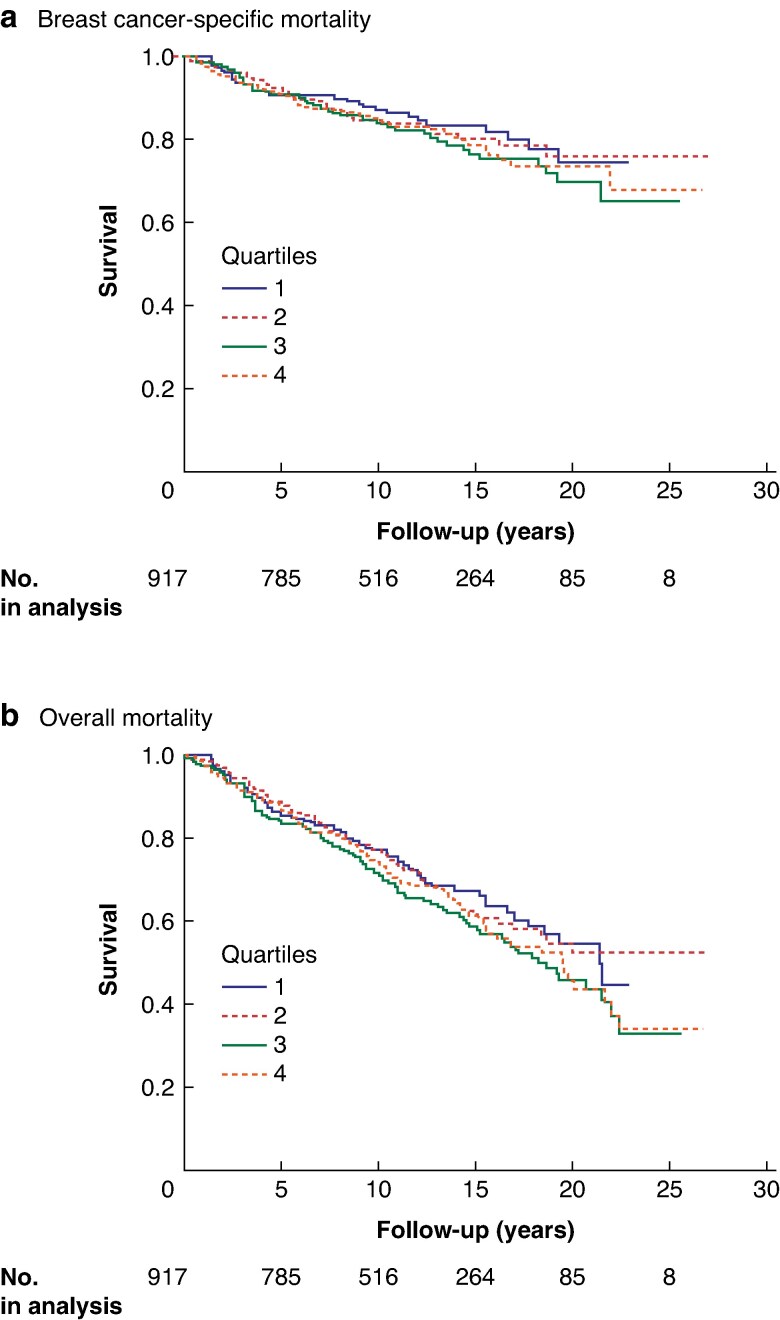
Kaplan–Meier plots demonstrating iodine levels (quartiles) in relation to breast cancer specific mortality and overall mortality **a** Breast cancer specific mortality, **b** overall mortality.

**Fig. 2:**
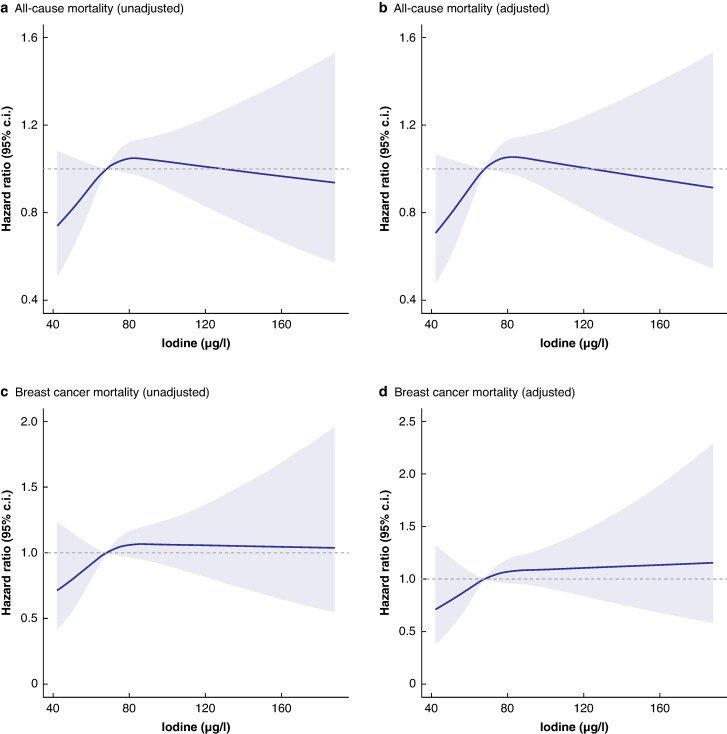
Restricted cubic spline analysis of breast cancer specific mortality and overall mortality; crude and adjusted analyses **a** All-cause mortality (unadjusted); non-linearity: *P* = 0.116, **b** all-cause mortality (adjusted); non-linearity: *P* = 0.094, **c** breast cancer mortality (unadjusted); non-linearity: *P* = 0.252, **d** breast cancer mortality (adjusted); non-linearity: *P* = 0.346. *P*-values from the likelihood test. The shaded areas represent 95% confidence intervals on the same scale as the y-axis. c.i., confidence interval.

Patients with high iodine levels (Q3 and Q4) showed a non-significant higher breast cancer specific mortality compared with patients with low levels (Q1): the adjusted HR for Q3 was 1.30 (95% c.i. 0.82 to 2.04) and in Q4 it was 1.26 (0.79 to 1.99) (*Table 3* and *Fig. 1*). The results for overall mortality were similar. There was no clear association with mortality in the analysis using iodine levels as a continuous variable (*Table 3*). To assess potential non-linear associations, restricted cubic splines models were computed (*Fig. 2*). There was no clear pattern, and none of the associations were non-linear (all *P* > 0.05).

**Table 3:** Breast cancer-specific and overall mortality in different iodine quartiles

Iodine quartile	No.	Breast cancer mortality				Overall mortality			
		Deaths (*n*)	Mortality/100 000 person-years	HR	HR*	Deaths (*n*)	Mortality/100 000 person-years	HR	HR*
1	231	35	1408	1	1	72	2896	1	1
2	227	39	1502	1.08 (0.69, 1.71)	1.04 (0.64, 1.67)	76	2927	1.00 (0.72, 1.38)	1.03 (0.74, 1.44)
3	231	47	1781	1.28 (0.82, 1.98)	1.30 (0.82, 2.04)	98	3714	1.26 (0.93, 1.71)	1.33 (0.97, 1.81)
4	229	45	1652	1.20 (0.77, 1.86)	1.26 (0.79, 1.99)	94	3452	1.17 (0.86, 1.59)	1.27 (0.92, 1.75)
Per SD	918	166		1.04 (0.91, 1.19)	1.06 (0.91, 1.24)	340		1.02 (0.92, 1.13)	1.02 (0.92, 1.14)
Total	918	166	1589			340	3255		

Values in parentheses are 95% confidence intervals. HR, hazard ratio; c.i., confidence interval; SD, standard deviation. *Adjusted for age at diagnosis, oestrogen receptor, progesterone receptor, human epidermal growth factor receptor, Ki67, histological grade, tumour size, lymph node metastases, and distant metastases.

**Fig. 3:**
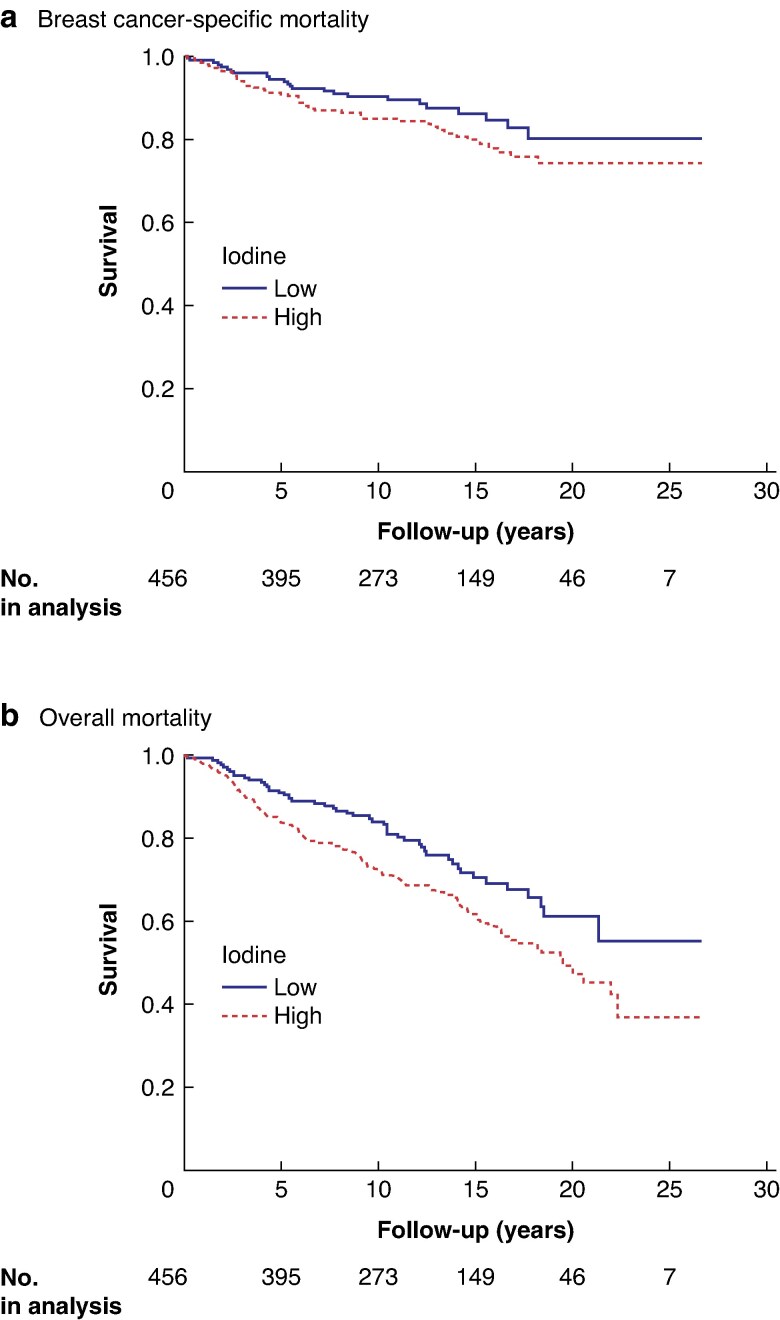
Kaplan–Meier plots demonstrating iodine levels (quartiles) in relation to breast cancer specific mortality and overall mortality in women with high selenium levels **a** Breast cancer specific mortality, **b** overall mortality.

When concentrations were dichotomized, patients with high iodine levels had an adjusted HR of 1.28 (95% c.i. 0.94 to 1.76) related to breast cancer-specific mortality (*Table 4*). The cut-off point for selenium and iodine dichotomization was 90.4 µg/l and 68 µg/l, respectively. The corresponding HR for overall mortality was 1.28 (1.03 to 1.59). The cohort was further stratified for serum selenium concentrations using dichotomization. In the group who had low selenium levels, HRs for iodine levels were close to unity. In the stratum with high selenium levels, women with high iodine concentrations had a statistically significant risk associated with breast cancer-specific mortality (adjusted HR 1.90, 1.09 to 3.32; *P* for interaction = 0.25), (*Table 4*, *Fig. 3*). The corresponding HR for overall mortality was 1.81 (1.26 to 2.61; *P* for interaction = 0.07).

**Table 4:** Breast cancer-specific and overall mortality in different iodine groups (dichotomized as high/low) stratified for selenium levels (high/low)

Selenium strata	Iodine levels	No.	Breast cancer-specific mortality			Overall mortality		
			Deaths (*n*)	HR	HR*	Deaths (*n*)	HR	HR*
All	Low iodine	458	74	1	1	148	1	1
All	High iodine	460	92	1.19 (0.87, 1.61)	1.28 (0.94, 1.76	192	1.22 (0.98, 1.51)	1.28 (1.03, 1.59)
Low selenium	Low iodine	261	49	1	1	97	1	1
Low selenium	High iodine	200	44	1.09 (0.73, 1.64)	0.99 (0.65, 1.53)	86	1.06 (0.79, 1.41)	0.99 (0.73, 1.34)
High selenium	Low iodine	197	25	1	1	51	1	1
High selenium	High iodine	260	48	1.46 (0.90, 2.36)	1.90 (1.09, 3.32)	106	1.55 (1.11, 2.16)	1.81 (1.26, 2.61)

Values in parentheses are 95% confidence intervals. HR, hazard ratio, c.i., confidence interval. *Adjusted for: age at diagnosis, oestrogen receptor, progesterone receptor, human epidermal growth factor receptor, Ki67, histological grade, tumour size, lymph node metastases, and distant metastases.

A similar analysis was performed to investigate how serum selenium, stratified for iodine, was associated with breast cancer-specific and overall mortality. High selenium levels were associated with a lower breast cancer-specific mortality; the adjusted HR was 0.69 (95% c.i. 0.50 to 0.95). The HR was similar in relation to overall mortality (*Table 5*). In the stratum with low iodine concentrations, the HRs for mortality were lower than in the stratum with high iodine concentrations. The *P-*values for interaction were as above, as the same models were used to calculate these values.

**Table 5:** Breast cancer-specific and overall mortality in different selenium groups (dichotomized as high/low) stratified for iodine levels (high/low)

Iodine strata	Selenium levels	No.	Breast cancer specific mortality			Overall mortality		
			Deaths (*n*)	HR	HR*	Deaths	HR	HR*
All	Low selenium	461	93	1	1	183	1	1
All	High selenium	460	73	0.73 (0.54, 1.00)	0.69 (0.50, 0.95)	157	0.79 (0.64, 0.97)	0.73 (0.58, 0.91)
Low iodine	Low selenium	261	49	1	1	97	1	1
Low iodine	High selenium	197	25	0.61 (0.38, 0.98)	0.52 (0.31, 0.88)	51	0.61 (0.44, 0.86)	0.50 (0.35, 0.72)
High iodine	Low selenium	200	44	1	1	86	1	1
High iodine	High selenium	260	48	0.81 (0.54, 1.21)	0.71 (0.46, 1.09)	106	0.90 (0.68, 1.19)	0.85 (0.63, 1.14)

Values in parentheses are 95% confidence intervals. HR, hazard ratio; c.i., confidence interval. *Adjusted for: age at diagnosis, oestrogen receptor, progesterone receptor, human epidermal growth factor receptor, Ki67, histological grade, tumour size, lymph node metastases, and distant metastases.

Sensitivity analyses excluding patients with missing information on tumour size were very similar to those including all patients (data not shown). Likewise, adjusting for storage time did not affect HRs to any considerable extent (data not shown).

## Discussion

The overall analysis indicated that high iodine levels may be associated with mortality following breast cancer. This association was statistically significant among women with high selenium levels. A previous study based on the same original cohort, the MDCS, has shown that high selenium levels are associated with a better breast cancer-specific survival and this indicates that iodine and selenium may interact, in terms of prognosis^13^.

Few studies have investigated the epidemiologic relationship between iodine and breast cancer survival. One previous study^3^ found that women with high iodine levels in combination with high selenium levels had a low risk of breast cancer. A case-control study^17^ in Korea did find an inverse relationship between seaweed intake, that is, a food item high in iodine, and breast cancer risk.

Similar theories have been suggested regarding breast cancer prognosis, indirectly by the observation of lower breast cancer death rates among the wives of fishermen in Norway^18^. A study^19^ from Spain showed a geographical correlation between high breast cancer mortality and iodine deficiency areas, suggesting a possible effect of iodine as a common denominator for breast cancer risk and/or survival. However, as the incidence in these studies is unknown, nothing can be concluded regarding whether a high or low exposure could have affected the risk of breast cancer or survival following the disease.

In Japan and Korea, iodine and selenium intake through diet is known to be high, and breast cancer incidence and mortality rates are also very low^20,21^. A nutritional study^22^ from Japan has observed low breast cancer and overall mortality among women with a moderate consumption of miso soup, a broth rich in iodine. Although iodine has also been proposed as having inhibitory effects on tumour progression in other cancers, a study^8,23^ based on the NHANES III did not find an association between urinary iodine concentration and overall cancer mortality. Previous findings^13,24^ regarding serum selenium and survival have found that high levels are associated with a low mortality, that is, a favourable survival.

There are some biological mechanisms of particular interest involved in iodine metabolism, which could play a role in breast cancer progression. The transport protein sodium/iodine symporter, which is involved in the cellular active transport of iodide, is absent in healthy breast tissue but has been seen in up to 80% of breast cancers^25^. There are several selenoenzymes that take part in iodine regulation, and one group responsible for activation/deactivation of thyroid hormone metabolism is the iodothyronine deiodinases. Thyroxine 5-deiodinase (coded by the gene *DIO3*) is responsible for deactivation of thyroid hormones and the authors’ group has previously reported that the expression of *DIO3* is associated with a poor prognosis following breast cancer; interestingly, specifically in women with high selenium. Contrary to this, the expression of *DIO1*, coding for selenoenzyme type I iodothyronine deiodinase, which catalyses the deiodination of T4 into T3, is associated with a favourable prognosis, specifically in women with high selenium^26^. These findings support the possibility that iodine and selenium interact in relation to breast cancer prognosis.

It has been also suggested that iodine itself could promote tumour progression by ER-mediated cancer cell growth and act as a generator of reactive oxygen species, offering a possible explanation for high mortality with high iodine values^27^. Even though this evidence does not directly reflect on how the exposure to iodine would affect prognosis following breast cancer, it does highlight the active participation of iodine and selenium in breast tumours.

Nevertheless, it has been shown that molecular iodine supplementation has noticeable inhibitory effects on tumour growth, invasiveness markers, and survival in animal experiments^1^. In a recent review^28^, Aceves and colleagues presented an explanatory model showing that it is the chemical form of molecular iodine I_2_, rather than iodide I^−^, that has antineoplastic effects on cancer cells without causing adverse effects on other organs sensitive to iodine excess. Thus, the distribution of the chemical form of iodine intake could play a role in its biological effects in the organism and could be a reason why previous experimental studies have seen both beneficial and harmful effects of iodine in relation to breast cancer.

Contrary to a previous study^3^, which demonstrated a risk reduction in patients having high iodine values, the present study shows high iodine in serum to be a marker for a poor prognosis following disease. This pattern has also been seen for other risk-modulating factors for breast cancer, that is, hormonal replacement therapy increases risk but may lead to a lower mortality, and breast feeding may decrease risk but could have a worse prognosis^29,30^. The reason for this is not clear, but the promotion of specific subgroups of tumours may contribute to this pattern. The mode of action of iodine in breast cancer is yet to be elucidated and there could be other causes for the result in the present study involving unknown biological processes.

There are several strengths to this study. The reliability of the measurements of iodine and selenium was high, with low inter-batch coefficient of variation values for the serum samples. Moreover, serum iodine reference ranges are not universally standardized, partially due to variability in laboratory methods, but also for practical reasons and limited available data. A Chinese peer-reviewed study^31^ has reported a population-derived reference range for serum iodine of 36.0–79.3 μg/l, whilst another study^32^ reported a mean(s.d.) of 63.78(18.92) μg/l for healthy adults. A meta-analysis^33^, also from China, reported a range as wide as 23.92–183.50 μg/l. For serum selenium reference values, there are more data available. A literature review^34^ found a range of 48.2 to 124.00 µg/l and a weighted mean(s.d.) of 85.19(14.58) µg/l for European healthy adults. This makes it unlikely that the results of this study are driven by abnormal or toxic overall levels.

The international standardized method for evaluating population iodine status is by measuring urinary iodine concentration^35^. However, this is an insensitive determinant for iodine status at an individual level due to high diurnal variability related directly to intake, with up to 90% being excreted in the urine. Uptake is further dependent on baseline iodine status due to up and down regulation of transport proteins; that is, in a state of iodine deficiency, uptake is increased and less is excreted. Serum levels, on the other hand, are considered to be more stable despite variations due to differences in intake and are therefore considered a better representation of biologically available amounts as a determinant of long-term exposure^36^. However, it is important to note that the measurements for this study are of total iodine and selenium present in serum and do not consider the distribution of the molecular forms as iodide or iodine, or selenium or selenoproteins.

Furthermore, the MDCS is a large, population-based, prospective cohort with a 43% participation rate among the invited women and is considered representative for the general population in Malmö in terms of risk factors and sociodemographic characteristics^12^.

Another strength is the high quality of the follow-up data used. The incident breast cancer cases from the MDCS were identified through cross-linkage with The Swedish Cancer Registry. The validity of The Swedish Cancer Registry is high with, only 1.6% underreporting for breast cancer^37^.

The Swedish Cause of Death Register was used to collect vital status and cause of death data. The register is almost complete, with 96% of the individuals having a registered cause of death. It has been shown to be correct in 90% of cases where malignancy was the cause of death^38^.

There are some limitations to this study. The measurements of serum values were only performed once, and hence variations in daily values are to some degree expected. Furthermore, the samples have been stored for over 20 years, and it is difficult to exclude the potential effect of evaporation or precipitation of the samples. To test for this, sensitivity analyses were performed, adjusting for storage time. This showed little or no effect on the results of the analyses, and hence the possibility of misclassification of exposure is considered to be rather small in this regard. The multivariable analyses included some covariables with missing values. There was no clear pattern of missingness related to iodine quartiles, and the sensitivity analysis excluding patients with missing information on tumour size did not suggest that it had affected the multivariable analyses.

Iodine was associated with a high mortality, specifically in women with high selenium levels. Iodine and selenium intakes have common sources, for example diets rich in seafood. A limitation is that there was no information on other heavy metals that may potentially be present in such diets, for example mercury, cadmium, lead, or arsenic. Iodine and selenium intake may be increased using dietary supplements. Unfortunately, there was no information on supplements for iodine, the main exposure in the present study. This is a limitation, but it can be expected that serum levels of iodine reflect both dietary intake and supplements. In this study it cannot be assessed whether there is any difference in activity from dietary iodine or selenium *versus* supplement use.

There was no adjustment for breast cancer treatment regimens, as patients treated during this time were offered standardized treatment options according to the tumour characteristics and stage.

High serum iodine levels were associated with a high mortality in women diagnosed with breast cancer. This association was particularly seen in those with high serum selenium. Serum selenium levels alone showed an inverse association with mortality. This is the first study to report on the association between iodine levels and breast cancer prognosis. Future studies should investigate this in relation to, for example, iodine receptors status and/or thyroid hormone levels.

## Data Availability

The data used for this study are available on reasonable request from the corresponding author.
